# BioM2MetDisease: a manually curated database for associations between microRNAs, metabolites, small molecules and metabolic diseases

**DOI:** 10.1093/database/bax037

**Published:** 2017-05-12

**Authors:** Yanjun Xu, Haixiu Yang, Tan Wu, Qun Dong, Zeguo Sun, Desi Shang, Feng Li, Yingqi Xu, Fei Su, Siyao Liu, Yunpeng Zhang, Xia Li

**Affiliations:** College of Bioinformatics Science and Technology, Harbin Medical University, Harbin, Heilongjiang 150081, China

## Abstract

BioM2MetDisease is a manually curated database that aims to provide a comprehensive and experimentally supported resource of associations between metabolic diseases and various biomolecules. Recently, metabolic diseases such as diabetes have become one of the leading threats to people’s health. Metabolic disease associated with alterations of multiple types of biomolecules such as miRNAs and metabolites. An integrated and high-quality data source that collection of metabolic disease associated biomolecules is essential for exploring the underlying molecular mechanisms and discovering novel therapeutics. Here, we developed the BioM2MetDisease database, which currently documents 2681 entries of relationships between 1147 biomolecules (miRNAs, metabolites and small molecules/drugs) and 78 metabolic diseases across 14 species. Each entry includes biomolecule category, species, biomolecule name, disease name, dysregulation pattern, experimental technique, a brief description of metabolic disease-biomolecule relationships, the reference, additional annotation information etc. BioM2MetDisease provides a user-friendly interface to explore and retrieve all data conveniently. A submission page was also offered for researchers to submit new associations between biomolecules and metabolic diseases. BioM2MetDisease provides a comprehensive resource for studying biology molecules act in metabolic diseases, and it is helpful for understanding the molecular mechanisms and developing novel therapeutics for metabolic diseases.

**Database URL:** http://www.bio-bigdata.com/BioM2MetDisease/

## Introduction

Metabolic diseases such as diabetes, obesity and metabolic syndrome, are chronic complex diseases and dominated by abnormal metabolism of body, have become one of the leading public health problem in the world (1, 2). One of the major challenges for researchers is to understand the underlying molecular mechanisms of metabolic diseases.

Metabolic diseases associated with the alteration of multiple types of biomolecules. Some well studied biomolecule classes include miRNAs, a class of small noncoding RNA molecules, which regulated its target mRNAs by binding to the seed matches of the target mRNA sequence and then degrade or inhibit the protein translation of target mRNAs at the post-transcriptional level (3). Currently, increasing evidences suggest that miRNAs play important roles in the initiation and progression of metabolic diseases (4–7). Also, metabolite has now been recognized as an important class of regulators in many physiological and pathophysiological processes such as cancer, diabetes and osteoporosis (8–11). They are also widely applied in discovery of diagnostic biomarkers as well as drug development and discovery (12–14). In addition, the ultimate objective of biomedical research is not only to reveal the underlie biomolecules of disease, but to find drugs that are used to treat them. For this purpose, a high-quality data source that stores metabolic diseases related small molecules/drugs is thus extremely beneficial for their therapeutics.

Due to the significant roles of these biomolecules, several data sources have been provided for storing their related information. For example, databases such as miRBase (15) and miRGen (16) focused on integrating sequences or annotation information of miRNAs. TargetScan (17), TarBase (18) and mirTarBase (19) provide computational prediction or experimentally validation of miRNA-target relationships. HMDD (20), miR2Disease (21) and phenomiR (22) contain experimentally supported miRNA and various disease associations. DIANA-miRPath identifies miRNA regulated pathways (23). HMDB (24) offers a comprehensive human metabolome data source including associations between metabolites and diseases. PubChem (25) is an online repository of resource about compound information including metabolites and small molecules (drugs). The above databases provide valuable aid for deciphering the functional roles of these biomolecules in diseases. But our knowledge about these biomolecules in metabolic diseases is still limited and few resource currently focus on collecting the latest associations between metabolic diseases and these biomolecules. Metabolic diseases such as obesity and diabetes with an increasing prevalence have drawn more and more attention (26). Currently, accumulating studies focused on investigating the activity of various biomolecules (miRNAs, metabolites and drugs, etc.) in metabolic diseases (5, 6, 8). However, these experimentally supported associations are scattered among the massive literatures, which is an obstruction for researchers to thoroughly understand the underlying molecular mechanisms and to find novel therapeutics of metabolic diseases. In particular, a comprehensive and high-quality data source that concentrates on metabolic diseases and devotes to integrate various related biomolecules and treatment drugs remains unavailable.

To fill this gap, we have developed the BioM2MetDisease database which collects experimental supported associations between biomolecules (miRNAs, metabolites, small molecules/drugs) and metabolic diseases. All associations in BioM2MetDisease were manually curated from literatures. In current release, BioM2MetDisease contains 2681 entries of relationships between 524 miRNAs and 45 metabolic diseases; 281 metabolites and 35 metabolic diseases; 349 small molecules/drugs and 57 metabolic diseases across 14 species. We hope that this database mainly developed for metabolic diseases could serve as an important resource for the research of metabolic disease in the future.

## Materials and methods

### Data collection

Entries describing the associations between various metabolic diseases and miRNAs, metabolites, small molecules/drugs, respectively, were all manually curated. First, we searched the PubMed database with key words including ‘miRNA’, ‘microRNA’, ‘metabolite’, ‘small molecule’ and ‘drug’ with a list of disease names (see Supplementary Table S1). Disease names shown in Supplementary Table S1 were involved in the ‘Endocrine, nutritional and metabolic diseases (E00-E90)’ class in International Classification of Diseases (ICD-10) or related with the metabolism disorder. >7000 literatures which published in the recent 5 years were returned. We selected publications that contain relationships between biomolecules (miRNAs, metabolites, small molecules/drugs) and metabolic diseases. Then, we manually curated the experimentally supported biomolecules (miRNAs, metabolites, small molecules/drugs)-metabolic diseases associations from these selected literatures. At the same time, we also extracted detailed information about the association including biomolecule (miRNA, metabolite, small molecule/drug) name, metabolic disease name, experimental techniques (e.g. microarray, northern blot, NMR), experimental tissue, dysregulation patterns (e.g. up-regulation, down-regulation), interaction gene symbol, species information, the reference information (PubMed ID, year of publication and reference title) and a brief description of the associations from the original literature. In this step, we only extracted experimentally supported biomolecule-metabolic diseases associations and referred to the strict criteria in previous studies (20, 21, 27, 28). Especially, in order to ensure the reliability of data, each extracted entry was checked by at least two researchers. We manually classified the associations into ‘low-throughput’ such as northern blot, PCR and ‘high-throughput’ such as microarray according to the confidence level of experimental technologies. In BioM2MetDisease database, approximately 75% entries were validated through low throughput experimental methods.

### Nomenclature standardization and classification

As detailed description of the associations were varied among different literatures such as the biomolecule names and disease names. To address this issue, we normalized the species names according to that of National Center for Biotechnology Information and replaced some of the biomolecule names as the commonly used one. We also used disease ontology (DO: http://disease-ontology.org) and a standardized classification scheme, ICD-10 classification to annotate each metabolic disease. To make the compounds and miRNAs consistent with other database, we provided the PubChem Compound Identifier (CID) for metabolites, small molecules/drugs in PubChem database (25) and miRBase (15) accession for miRNAs.

### Database framework and web interface

Data stored in the BioM2MetDisease database were managed by using MySQL. The web server of BioM2MetDisease was developed based on Java. The BioM2MetDisease database is freely available at http://www.bio-bigdata.com/BioM2MetDisease/.

## Results

### Database content

**Figure 1.**
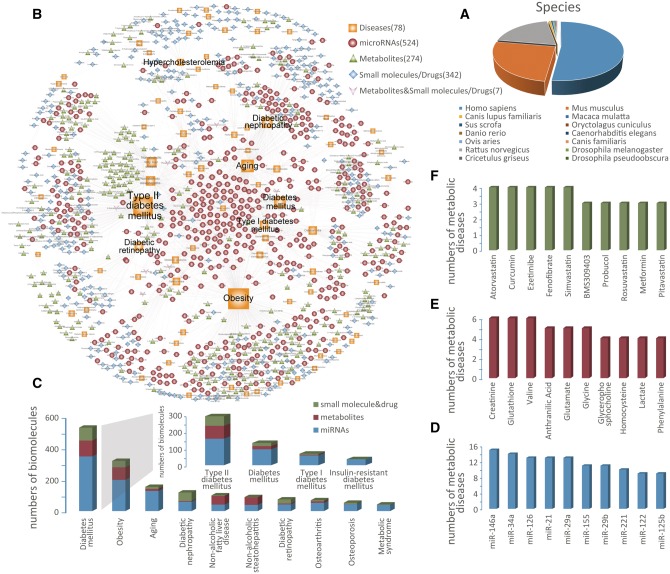
Network and distribution of data in BioM2MetDisease. (**A**) Statistics and distributions of species. (**B**) The biomolecule-metabolic disease bipartite network. Nodes correspond to biomolecules (miRNAs, metabolites, small molecules/drugs) and metabolic diseases, and the edges correspond to experimentally supported associations. The size of the nodes corresponds to the nodes’ degree. Distributions of the top ten high connectivity nodes of each category: metabolic diseases (**C**), miRNAs (**D**), metabolites (**E**) and small molecules/drugs (**F**).

To gain the understanding of association between biomolecules and metabolic diseases, BioM2MetDisease provides detailed information including biomolecule and metabolic disease names, species, experimental techniques, experimental tissue, dysregulation patterns, etc. to describe how a biomolecule is related to metabolic disease (see materials and methods). BioM2MetDisease also offers PubChem CID for metabolites and small molecules/drugs, miRBase accession for miRNAs, DO identifier and ICD-10 classification for metabolic diseases. In addition, we also provided convenient links to other related databases including miRNAs in miRBase (15), metabolites in HMDB (24) and PubChem (25), small molecules/drugs in DrugBank (29) and PubChem (25). A hyper link to the original reference in the PubMed database through official PubMed ID was also offered for each entry. In total, 2681 entries of relationships between 1147 biomolecules (miRNAs, metabolites and small molecules/drugs) and 78 metabolic diseases across 14 species from nearly 1300 literatures were manually curated. These entries contain experimentally supported associations between 524 miRNAs and 45 metabolic diseases; 281 metabolites and 35 metabolic diseases; 349 small molecules/drugs and 57 metabolic diseases. Here, the same biomolecules in different species were counted only once. The homo sapiens, mus musculus and rattus norvegicus were the top three species (Figure 1A).

By exploring the data from BioM2MetDisease, we found some important aspects. We constructed a bipartite network with various metabolic diseases and biomolecules (miRNAs, metabolites, small molecules/drugs) as node (Figure 1B). Nodes with high connectivity in a network are the central part of network, important to the stability of biological system (30, 31). We found that diabetes mellitus, obesity, aging, osteoporosis and metabolic syndrome which share high connectivity, are associated with 530, 319, 153, 53 and 42 biomolecules respectively (Figure 1C). Here, the species information was not considered. For biomolecules, miR-146a, miR-34a and miR-21 which have high connectivity were connected with 15, 14, 13 metabolic diseases, respectively, (Figure 1D), while Creatinine, Glutathione and Valine are three of the most highly connected nodes in metabolites (Figure 1E). These indicate the importance of these biomolecules in metabolic diseases. One should notice that the number of related metabolic diseases of miRNAs is higher than that of metabolites (Wilcoxon test: *P*-value = 0.00023), which may due to the preference of miRNAs studies. Most of small molecules/drugs that with the high connectivity belongs to lipid/cholesterol-lowering drugs (e.g. atorvastatin, simvastatin and fenofibrate) (Figure 1F). This suggest that higher level of lipid/cholesterol may be an important clinical indication of metabolic diseases.

#### Database use and access

##### User interface

**Figure 2.**
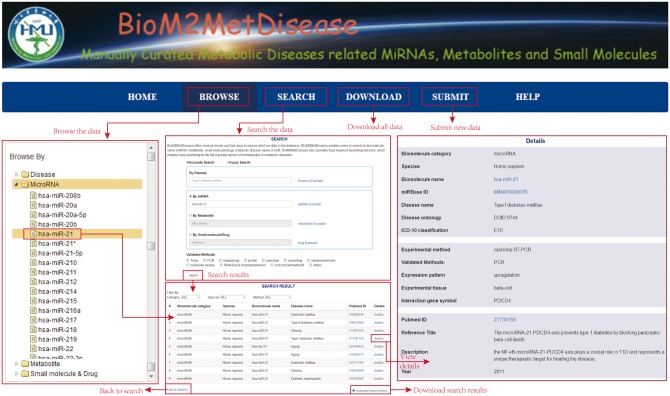
A schematic workﬂow of BioM2MetDisease.

BioM2MetDisease provides a user-friendly interface that enable users to browse, search and retrieve all entries in database (Figure 2). In the ‘Browse’ page, users can browse the biomolecule–metabolic disease associations by clicking a specific biomolecule (miRNA, metabolite, small molecule/drug) or metabolic disease name. Then, a list of matched entries is returned. Users can filter the entries by selecting a specific species, experimental method class and biomolecule category, etc. For example, users can retrieve more reliable relationships by selecting the ‘low-throughput’ option.

In the ‘Search’ page, users can search by inputting a specific metabolic disease name, a biomolecule (miRNA, metabolite or small molecule/drug) name or both. BioM2MetDisease offers a ‘fuzzy search’ function for the entries by giving the full or partial names of biomolecules or metabolic diseases in the ‘Search’ page. The ‘Search’ is case-insensitive. Furthermore, BioM2MetDisease offers an option in the ‘Search’ page that enables users to filter associations by specific experimental methods. In the search result page, BioM2MetDisease provides options for users to filter associations by biomolecule category, species, detection method, etc. Users can select associations according to the level of confidence of experimental outcomes by clicking the ‘method’ option which includes ‘high-throughput’ and ‘low-throughput’.

BioM2MetDisease provides a submission page for users to submit novelty validated associations. Once the submission approved by our review committee, the record(s) will be integrated in BioM2MetDisease. In addition, BioM2MetDisease provides a download page that enables users to freely download all associations stored in the database. A detailed tutorial for users is also offered in the ‘Help’ page.

##### Cases application of BioM2MetDisease

To exemplify the usage of BioM2MetDisease database and the advanced features of it for biomolecules and metabolite diseases studies, we firstly input ‘miR-34a’ as the miRNA name on the search page. After clicking the ‘Search’ button, the result page shown and displayed that most records of miR-34a were ‘up-regulated’. Particularly, the recorded entries consistently shown that miR-34a is ‘up-regulated’ in type II diabetes mellitus. This indicates that higher expression level of miR-34a may promote the development of metabolic diseases.

BioM2MetDisease offers the potential mechanisms and therapeutics for various metabolic diseases and also the functional information of biomolecules. For example, we then input ‘type II diabetes mellitus’ as disease name in search page, we found that many miRNAs and metabolites such as hsa-miR-21 and hsa-miR-181a were dysregulation in human type II diabetes mellitus (T2D). The functional description in BioM2Metadisease shown that up-regulation of miR-181a plays a role to link between adipose tissue dysfunction and T2D. Furthermore, overexpression of miR-181a decreases levels and activity of SIRT1 protein, and causes insulin resistance in hepatic cells, which is important for development of T2D (32–34). In addition, several small molecules/drugs that are already in use for therapeutics of T2D or novelty identified based on relevant clinical studies (including positive, negative and ongoing trials) and/or animal models were retrieved in BioM2Metdisease, such as metformin and HD0471042.

Information in the BioM2MetDisease can be used to describe the relationships among different metabolic diseases. For example, by inputting ‘metformin’, a first-line drug of treatment T2D, the results shown that it is connected with three metabolic diseases including two diabetes mellitus related diseases (diabetic nephropathy and T2D) and obesity. A closer inspection of the data in BioM2MetDisease found that obesity and T2D share up to 95 biomolecules, which reveals evidence for supporting the highly association of these two metabolic diseases (35–37).

By querying ‘methylglyoxal’ as the metabolite name, we found that it is associated with three metabolic diseases (type II diabetes mellitus, diabetic retinopathy and obesity) in BioM2Metdisease. However, the only related metabolic disease of it documented in HMDB (24) is diabetes mellitus. In short, BioM2MetDisease can serve as a publicly available integrated data source to study the functions and mechanisms of various biomolecules in metabolic diseases and provide potential novel therapeutics.

### Future extensions

Metabolic diseases, such as diabetes, have now developed into a world-wide prevalent diseases, and increasing number of studies focused on the metabolic disease (26). Thus, the number of validated metabolic diseases associated biomolecules will continue to increase in the future. We will collect interested literatures by searching on PubMed with these key words periodically in the future. The database will update annually based on the availability of literatures from new studies. We will incorporate more classes of biomolecules such as protein, lncRNA, circular RNA, etc. as well as more data sources to increase the content coverage of BioM2MetDisease and to make it more comprehensive. In addition, to facilitate the utility of the database, online network analysis, visualization and functional annotation tools will be integrated in the future.

## Discussion and conclusion

Metabolic disease has emerged as a major public health problem in the world wide. Emerging evidence suggest that metabolic disease is related with the aberrant level of multiple type biomolecules. A collection of metabolic diseases related biomolecules is important for the researches in this field. Thus, we developed BioM2MetDisease, a database aims to provide a central resource for biologists who devote to studies of metabolic diseases. BioM2MetDisease is a comprehensive resource, in which approximately 2700 experimentally supported associations between 78 metabolic diseases and 1147 biomolecules across 14 species were collected. Currently, some high-quality data sources such as miR2Disease (21), phenomiR (38) and HMDD (20) store a number of disease associated miRNAs, and HMDB (24) contains metabolite-disease associations. However, these databases were not specifically designed for metabolic diseases and lacked the content coverage in terms of the metabolic disease types, classes of biomolecules and species. For example, miR2Disease only contains miRNA and disease associations in Homo sapiens (21). We further compared miR2Disease, phenomiR and HMDD databases with BioM2MetDisease in terms of the amount of metabolic diseases. We found that BioM2MetDisease contains much more metabolic diseases than other three databases (Supplementary Figure S1). In the current release, biomolecules including miRNAs, metabolites and small molecules/drugs were collected, and more types of biomolecules will be incorporated in the future.

Overall, BioM2MetDisease not only offers a comprehensive resource of biomolecule-metabolic disease associations, but also provides a global insight into biomolecules in various metabolic diseases. We believe that BioM2MetDisease will be a valuable resource for biologists to explore the pathogenesis of metabolic diseases and develop new therapeutics.

## Funding

The National Program on Key Basic Research Project [973 Program, Grant Nos. 2014CB910504], the National High Technology Research and Development Program of China [863 Program, Grant Nos. 2014AA021102] (in part), the National Natural Science Foundation of China [Grant Nos. 91439117, 61473106, 31501074, and 61603116]. 

*Conﬂict of interest.* None declared.

## Supplementary data

Supplementary data are available at *Database* Online.

## Supplementary Material

Supplementary Data
