# Supplementary material for: BioM2MetDisease: a manually curated database for associations between microRNAs, metabolites, small molecules and metabolic diseases
Source: Database (Oxford). 2017 May 12;2017:bax037. doi: 10.1093/database/bax037 (PMC5467570; doi:10.1093/database/bax037)
Supplement: Supplementary Data [file bax037_Supp.docx]

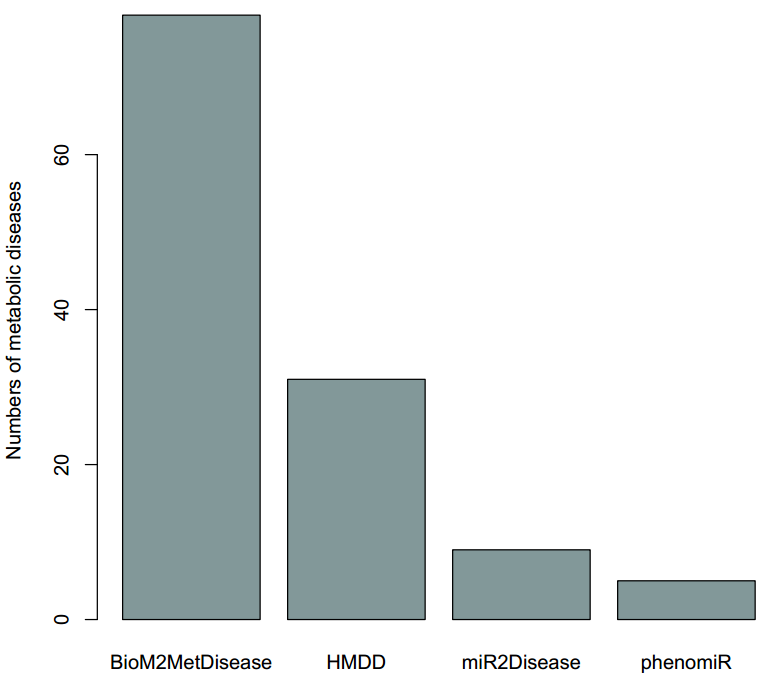


**Figure S1.** The amount of metabolic diseases involved in BioM2MetDisease, HMDD, miR2Disease and phenomiR.

**Table S1. The name list of metabolic diseases as keyword searched in PubMed**

| **The name of metabolic diseases** |
| --- |
| 2-Methyl-3-hydroxybutyryl-CoA dehydrogenase (MHBD) deficiency |
| 3-Hydroxy-3-methylglutaryl-CoA lyase deficiency |
| 3-Hydroxyacyl-CoA dehydrogenase deficiency |
| 3-Methylcrotonylglycinuria |
| 3-Methylglutaconic aciduria (MGCA) |
| 3-Phosphoglycerate dehydrogenase (3-PGDH) deficiency |
| 46,XX disorders of sex development (Disorders related to androgen excess) |
| 46,XY disorders of sex development (Disorders in androgen synthesis or action) |
| Abetalipoproteinemia |
| Abnormal thyroid hormone metabolism |
| Acatalasia |
| Achalasia-Addisonianism-Alacrima synrrome |
| Acid phosphatase deficiency |
| Acne |
| Acne vulgaris |
| Acquired generalized lipodystrophy (AGL) |
| Acrodermatitis enteropathica |
| Acromegaly |
| Acute gouty arthritis |
| Adenylosuccinate lyase deficiency |
| Adrenocorticotropic hormone deficiency |
| Adrenoleukodystrophy |
| Adrenoleukodystrophy, neonatal (NALD) |
| Adult-onset autosomal dominant leukodystrophy (ADLD) |
| adult-onset type II citrullinemia |
| Aging |
| AICA-ribosiduria |
| Albuminuria |
| Aldosterone synthase deficiency |
| Alkaptonuria |
| Allan-Herndon-Dudley syndrome |
| alpha-Mannosidosis |
| Alpha-methylacetoacetic aciduria |
| Alpha-N-acetylgalactosaminidase deficiency |
| Alport's syndrome |
| Alstrom syndrome (AS) |
| Alzheimer's disease |
| Apparent mineralocorticoid excess syndrome |
| Argininosuccinic aciduria (ARGINSA) |
| Aristolochic acid nephropathy |
| Aromatase excess syndrome |
| Aspartylglucosaminuria |
| Ataxia with isolated vitamin E deficiency (AVED) |
| Atherosclerosis |
| Bamforth-Lazarus syndrome |
| Barth syndrome (BTHS) |
| Bartter syndrome |
| beta-Mannosidosis |
| Beta-ureidopropionase deficiency |
| Biotinidase deficiency |
| Brunner syndrome |
| Canavan disease |
| Carbamoyl phosphate synthetase I deficiency |
| Cerebrotendinous xanthomatosis |
| Choreoathetosis, hypothyroidism, and neonatal respiratory distress |
| Chylomicron retention disease (CRD) |
| Citrullinemia |
| cobalamin deficiency type C (cblC) |
| Combined lipase deficiency (CLD) |
| Combined SAP deficiency |
| Congenital adrenal hyperplasia |
| Congenital disorders of glycosylation (CDG) type I |
| Congenital disorders of glycosylation (CDG) type II |
| Congenital generalized lipodystrophy (CGL) |
| Congenital glucose-galactose malabsorption (GGM) |
| Congenital hyperthyroidism |
| Congenital lactase deficiency |
| Congenital nongoitrous hypothyroidism (CHNG) |
| Congenital sucrase-isomaltase deficiency |
| Congenital systemic glutamine deficiency (CSGD) |
| Corticosteroid-binding globulin (CBG) deficiency |
| Cortisone reductase deficiency (CRD) |
| Creatine deficiency syndrome |
| Cushing's syndrome |
| Cutis laxa |
| cystathionine β-synthase (CBS) deficiency |
| Cystathioninuria |
| Cystic fibrosis |
| Cystic leukoencephalopathy without megalencephaly |
| Cystinosis |
| Cystinuria |
| Danon disease |
| Defects in RecQ helicases |
| Defects in the degradation of ganglioside |
| Defects in the degradation of sphingomyelin |
| Defects in the degradation of sulfatide |
| Diabetes-related atherosclerosis |
| Diabetes-related cognitive dysfunction |
| Diabetes-related microvascular disease |
| Diabetes mellitus |
| Diabetic atherosclerosis |
| Diabetic cardiomyopathy |
| Diabetic coma |
| Diabetic macular edema |
| Diabetic microangiopathy |
| Diabetic nephropathy |
| Diabetic neuropathy |
| Diabetic retinopathy |
| Diabetic vascular disease |
| Dihydropyrimidine dehydrogenase deficiency |
| Dimethylglycine dehydrogenase deficiency (DMGDHD) |
| Diseases of the tricarboxylic acid cycle |
| Disorders of fatty-acid oxidation |
| Dorfman-Chanarin syndrome |
| Dyslipidemia |
| Dyslipidemic obesity |
| Erythropoietic porphyria |
| Fabry disease |
| Fabry nephropathy |
| Familial amyloidosis |
| Familial combined hyperlipidemia |
| Familial glucocorticoid deficiency (FGD) |
| Familial hyperinsulinemic hypoglycemia (HHF) |
| Familial hypobetalipoproteinemia (FHBL) |
| Familial hypocalciuric hypercalcemia (FHH1) |
| Familial Mediterranean fever |
| Familial partial lipodystrophy (FPL) |
| Familial renal glucosuria (FRG) |
| Fanconi renotubular syndrome (FTS) |
| Farber lipogranulomatosis |
| Fatty liver disease |
| Formiminotransferase deficiency |
| Fructose-1,6-bisphosphatase deficiency |
| Fucosidosis |
| GABA-transaminase deficiency |
| Galactosemia |
| Galactosialidosis |
| Gangliosidosis |
| Gaucher disease |
| Gestational diabetes mellitus |
| Gitelman syndrome |
| Globoid leukodystrophy |
| Glucocorticoid-remediable aldosteronism (GRA) |
| Glutaric acidemia |
| Glutaric aciduria type 1 |
| Glycerol kinase deficiency (GKD) |
| Glycogen storage diseases |
| Glycoproteinoses |
| Glycosylphosphatidylinositol (GPI) deficiency |
| GM1 gangliosidosis |
| GM2 gangliosidoses |
| Gout |
| Graves' disease |
| Growth delay due to insulin-like growth factor I resistance |
| Growth hormone insensitivity with immunodeficiency |
| Guanidinoacetate methyltransferase (GAMT) deficiency |
| Hartnup disorder |
| Hashimoto's thyroiditis |
| Hemochromatosis |
| Hepatic porphyria |
| Hereditary fructose intolerance |
| Hermansky-Pudlak syndrome |
| Histidinemia |
| HMG-CoA synthase (HMGCS) deficiency |
| Holocarboxylase synthetase deficiency |
| Homocystinuria |
| Huntington's disease |
| Hutchinson-Gilford progeria syndrome |
| Hyperaldosteronism |
| Hyperalphalipoproteinemia |
| Hyperammonemia |
| Hyperargininemia |
| Hyperbilirubinemia |
| Hypercalcemia infantile |
| Hypercarotenemia and vitamin A deficiency |
| Hypercholesterolemia |
| Hyperglucagonemia |
| Hyperglycemia |
| Hyperinsulinemia |
| Hyperlipidemia |
| Hyperlipoproteinemia, type I |
| Hyperlipoproteinemia, type III |
| Hyperlipoproteinemia, type V |
| Hyperlysinemia |
| Hypermethioninemia |
| Hyperornithinemia |
| Hyperornithinemia-hyperammonemia-homocitrullinuria syndrome |
| Hyperprolactinemia |
| Hyperprolinemia |
| Hyperthyroidism |
| Hypertriglyceridemia |
| Hyperuricemia |
| Hypoalphalipoproteinemia |
| Hypocalcemia |
| Hypoglycemic |
| Hypogonadotropic hypogonadism |
| Hypomagnesemia |
| Hypoparathyroidism |
| Hypophosphatasia |
| Hypophosphatemic osteomalacia |
| Hypophosphatemic rickets |
| Hypothyroidism |
| IgA nephropathy |
| Iminoglycinuria |
| Insulin-resistant diabetes mellitus |
| Insulin-resistant diabetes mellitus with acanthosis nigricans (IRAN) |
| Isobutyryl-CoA dehydrogenase (IBD) deficiency |
| Isolated follicle-stimulating hormone deficiency (IFSHD) |
| Isolated short stature |
| Isovaleric acidemia |
| Ketosis |
| Krabbe disease |
| LDL Receptor Disorder |
| Lecithin:cholesterol acyltransferase deficiency |
| Leprechaunism |
| Lesch-Nyhan syndrome |
| Leukodystrophy |
| Leukoencephalopathy with brain stem and spinal cord involvement and lactate elevation (LBSL) |
| Leukoencephalopathy with dystonia and motor neuropathy |
| Leukoencephalopathy with vanishing white matter |
| Lipodystrophy |
| Lipoid proteinosis |
| Lowe syndrome |
| Lysinuric protein intolerance (LPI) |
| Lysosomal acid lipase deficiency |
| Malonyl-CoA decarboxylase deficiency |
| Maple syrup urine disease |
| Maturity onset diabetes of the young (MODY) |
| Megalencephalic leukoencephalopathy with subcortical cysts (MLC) |
| Menkes disease |
| Metabolic syndrome |
| Metachromatic leukodystrophy |
| Methacrylic aciduria |
| Methylcobalamin deficiency type G (cblG) |
| Methylmalonate semialdehyde dehydrogenase (MMSDH) deficiency |
| Methylmalonic acidemia |
| Methylmalonic aciduria |
| Mitochondrial DNA depletion syndrome (MDS) |
| Mitochondrial dysfunction |
| Mitochondrial respiratory chain deficiencies (MRCD) |
| Mitochondrial trifunctional protein (TFP) deficiency |
| Muckle-Wells syndrome |
| Mucolipidosis II and III |
| Mucolipidosis IV |
| Mucopolysaccharidosis (MPS) |
| Mucopolysaccharidosis type I (MPS1) |
| Mucopolysaccharidosis type II (MPS2) |
| Mucopolysaccharidosis type III (MPS3) |
| Mucopolysaccharidosis type IV (MPS4) |
| Mucopolysaccharidosis type IX (MPS9) |
| Mucopolysaccharidosis type VI (MPS6) |
| Mucopolysaccharidosis type VII (MPS7) |
| Multiple sulfatase deficiency |
| N-acetylglutamate synthase (NAGS) deficiency |
| Nephrogenic syndrome of inappropriate antidiuresis (NSIAD) |
| Neurohypophyseal diabetes insipidus (NPDI) |
| Neuronal ceroid lipofuscinosis |
| Neutral lipid storage disease with myopathy |
| Niemann-Pick disease |
| Niemann-Pick disease type A |
| Niemann-Pick disease type B |
| Niemann-Pick disease type C |
| Non-alcoholic fatty liver disease |
| Non-alcoholic steatohepatitis |
| Non-insulin-dependent diabetes mellitus |
| Nonketotic hyperglycinemia |
| Obesity |
| Obesity-associated cardiovascular diseases |
| Obesity-induced diabetes |
| Obesity-related inflammation |
| Ocular albinism |
| Oculocutaneous albinism |
| Ornithinaemia |
| Ornithine transcarbamylase deficiency |
| Osteoarthritis |
| Osteomalacia |
| Osteoporosis |
| Parkinson's disease |
| Pelizaeus-Merzbacher disease |
| Pentosuria |
| Peroxisomal beta-oxidation enzyme deficiency |
| Phenylketonuria |
| Photoaging |
| Piebaldism |
| pigmented micronodular adrenocortical disease (PPNAD) |
| Pituitary Dwarfism (PD) |
| POLG related disorders |
| Polycystic lipomembranous osteodysplasia with sclerosing leukoencephalopathy (PLOSL) |
| Postmenopausal osteoporosis |
| Precocious puberty |
| Premature ovarian failure |
| Primary aldosteronism |
| Primary hyperammonemic disorders |
| Primary hyperoxaluria |
| Primary hyperparathyroidism |
| Prolidase deficiency (PD) |
| Propionic acidemia |
| Propionic aciduria |
| Proteinuria |
| Pseudohypoparathyroidism |
| Pycnodysostosis |
| Pyruvate carboxylase deficiency |
| Pyruvate dehydrogenase complex deficiency |
| Rabson-Mendenhall syndrome |
| Rickets |
| Saccharopinuria |
| Secondary hyperammonemia |
| Sialidosis |
| Sialuria/ Sialic acid storage disease |
| Sitosterolemia |
| Sjogren-Larsson syndrome |
| Steatohepatitis |
| Succinic semialdehyde dehydrogenase (SSADH) deficiency |
| Succinyl CoA:3-oxoacid CoA transferase (SCOT) deficiency |
| Sulfite oxidase deficiency |
| Tangier disease |
| Thiopurine S-methyltransferase deficiency (TPMT deficiency) |
| Thyroid dyshormonogenesis |
| Thyroid hormone resistance syndrome |
| Transaldolase (TALDO) deficiency |
| Trimethylaminuria (TMAU) |
| Type I diabetes mellitus |
| type I tyrosinemia |
| Type II diabetes mellitus |
| Tyrosinemia |
| Urocanase deficiency |
| vitamin A deficiency |
| Vitamin D-dependent rickets |
| vitamin D deficiency |
| vitamin E deficiency |
| Waardenburg syndrome (WS) |
| Weight loss |
| Wilson disease |
| Wolcott-Rallison syndrome |
| Wolfram syndrome (WFS) |
| Xanthinuria |
